# Neonatal intensive care unit phthalate exposure and preterm infant neurobehavioral performance

**DOI:** 10.1371/journal.pone.0193835

**Published:** 2018-03-05

**Authors:** Annemarie Stroustrup, Jennifer B. Bragg, Syam S. Andra, Paul C. Curtin, Emily A. Spear, Denise B. Sison, Allan C. Just, Manish Arora, Chris Gennings

**Affiliations:** 1 Division of Newborn Medicine, Department of Pediatrics, Icahn School of Medicine at Mount Sinai, New York, New York, United States of America; 2 Department of Obstetrics, Gynecology and Reproductive Science, Icahn School of Medicine at Mount Sinai, New York, New York, United States of America; 3 Department of Environmental Medicine and Public Health, Icahn School of Medicine at Mount Sinai, New York, New York, United States of America; University of Missouri Columbia, UNITED STATES

## Abstract

Every year in the United States, more than 300,000 infants are admitted to neonatal intensive care units (NICU) where they are exposed to a chemical-intensive hospital environment during a developmentally vulnerable period. The neurodevelopmental impact of environmental exposure to phthalates during the NICU stay is unknown. As phthalate exposure during the third trimester developmental window has been implicated in neurobehavioral deficits in term-born children that are strikingly similar to a phenotype of neurobehavioral morbidity common among children born premature, the role of early-life phthalate exposure on the neurodevelopmental trajectory of premature infants may be clinically important. In this study, premature newborns with birth weight <1500g were recruited to participate in a prospective environmental health cohort study, NICU-HEALTH (Hospital Exposures and Long-Term Health), part of the DINE (Developmental Impact of NICU Exposures) cohort of the ECHO (Environmental influences on Child Health Outcomes) program. Seventy-six percent of eligible infants enrolled in the study. Sixty-four of 81 infants survived and are included in this analysis. 164 urine specimens were analyzed for phthalate metabolites using high-performance liquid chromatography/tandem mass spectrometry. The NICU Network Neurobehavioral Scale (NNNS) was performed prior to NICU discharge. Linear and weighted quantile sum regression quantified associations between phthalate biomarkers and NNNS performance, and between phthalate biomarkers and intensity of medical intervention. The sum of di(2-ethylhexyl) phthalate metabolites (∑DEHP) was associated with *improved* performance on the Attention and Regulation scales. Specific mixtures of phthalate biomarkers were also associated with *improved* NNNS performance. More intense medical intervention was associated with higher ∑DEHP exposure. NICU-based exposure to phthalates mixtures was associated with improved attention and social response. This suggests that the impact of phthalate exposure on neurodevelopment may follow a non-linear trajectory, perhaps accelerating the development of certain neural networks. The long-term neurodevelopmental impact of NICU-based phthalate exposure needs to be evaluated.

## Introduction

Every year in the United States, more than 300,000 infants are admitted to neonatal intensive care units (NICUs) where, in addition to experiencing life-saving treatments, they are exposed to a chemical-intensive hospital environment during a developmentally vulnerable period.[1–5] Prematurity, particularly birth at gestational age < 32 weeks and weight < 1500g, is associated with a particular behavioral phenotype characterized by inattention, anxiety, and socialization difficulties[6] that can be measured as early as the toddler years.[7] Alterations in the developmental trajectory of the cerebral cortex—as opposed to focal brain injury—are thought to lead to behavioral morbidities associated with preterm birth.[6, 8] Prematurity-related impairments such as cognitive dysfunction, fine and gross motor impairments, attention deficit, hyperactivity, and autism spectrum disorders[6, 7, 9–18] are only partially explained by degree of prematurity or severity of illness in infancy.[19, 20] Traditional perinatal risk factors such as gestational age are not as strongly prognostic of neurodevelopmental outcome as previously thought.[21] Exposure to chemical toxicants during early development, notably exposure to phthalates, is implicated in alterations in neurodevelopment in term-born human and animal studies.[22, 23] Phthalates, chemical plasticizers known to leach out of medical equipment used to care for infants,[24, 25] are known to be present in biospecimens taken from NICU inpatients.[26–28] Whether hospital-based exposure to phthalates influences behavioral development of NICU graduates has not been evaluated previously.

Phthalates are a family of industrial chemicals non-covalently bonded to plastic materials to enhance flexibility and durability.[29] Particularly in conditions of elevated heat or humidity (present in neonatal incubators and respiratory support circuits), phthalates leach from the solid matrix because they are not chemically integral to the plastic scaffold.[24, 25] In the body, parent phthalate diesters undergo hydrolysis and glucuronidation, followed by excretion of monoester metabolite(s) in urine and stool.[30] Both parent diesters and monoester metabolites are biologically active.[31] Phthalate exposure can occur through ingestion, inhalation, dermal absorption, and parenteral routes.[29] Di(2-ethylhexyl) phthalate (DEHP) exposures in NICU infants may be 4,000 to 160,000 times higher than levels associated with toxicity.[32]

Associations between prenatal or early childhood exposure to phthalates and alterations in multiple domains of neurodevelopment have been reported in non-NICU populations.[33–39] Elevated *in utero* exposure to phthalate mixtures has been associated with poorer infant executive function, attention, and motor reflexes.[40, 41] *In utero* exposure to DEHP, the phthalate most commonly associated with polyvinyl chloride (PVC) plastic medical equipment, has been associated independently with childhood impairments in cognitive, motor, and executive function, as well as with hyperactivity, poor attention,[35, 42] and autism spectrum behaviors[43] in term-born cohorts.[35, 39, 44, 45] No biomarker-based study of preterm infants or preterm animal models during the “third trimester” developmental period between birth and term-equivalent has assessed the impact of phthalate exposure on neurodevelopmental outcomes.

We hypothesize that NICU-based exposure to phthalates contributes to altered behavioral development in preterm infants.[46] This hypothesis draws on noted similarities between abnormal attention and social interaction phenotypes that have been linked to *in utero* phthalate exposure in term cohorts[33–36, 40, 42] and the characteristic “preterm behavioral phenotype” described by Montagna and Nosarti[7]. Additionally, through data gathered in this study we preliminarily explore the hypothesis first investigated by Green *et al*.[27] that clinically-relevant phthalate exposure can be linked to specific NICU equipment.

## Materials and methods

### Participant identification and enrollment

Between 2011 and 2013, very low birth weight infants (VLBW; those with birth weight < 1500g) were recruited upon admission to the Mount Sinai Hospital NICU to participate in phase I of the NICU-HEALTH (Hospital Exposures and Long-Term Health) study, part of the DINE (Developmental Impact of NICU Exposures) cohort included in the ECHO (Environmental influences on Child Health Outcomes) program (ClinicalTrials.gov NCT01420029, NCT01963065, NCT03061890; http://www.nih.gov/echo). Birth weight rather than gestational age was used as an objective entry criterion to define our NICU-based preterm population. Gestational age may be inaccurate in patients without complete early prenatal care. Out-born infants (those transferred to our hospital after birth) and those with congenital anomalies or genetic syndromes were excluded. VLBW infants typically spend significant time in the NICU during a critical period of neurodevelopment. Written informed consent to participate in the study was obtained from participants’ parents prior to enrollment. The institutional review board of the Icahn School of Medicine at Mount Sinai approved the study.

### Specimen collection

Urine was collected by trained study staff between the week of birth and 34 weeks postmenstrual age (PMA) using methods adapted from Green[27] and Weuve.[28] The first specimen was collected during the first week of life. Initially, the study protocol called for collection of 3 urine specimens spanning the NICU hospitalization. After an initial start-up period, urine specimens were collected on a weekly basis throughout the NICU stay. Cotton balls were placed in the infant diaper and retrieved three hours later. Urine was squeezed from cotton not contaminated with stool. If stool contamination occurred, urine collection was re-attempted for up to three days until a clean specimen was obtained. As preterm infants do not stool as regularly as full-term infants, this approach was successful in most cases. Specimens were immediately refrigerated on the unit, then frozen within 6 hours at -80°C pending batch analysis. Urine specific gravity was measured by refractometry (Atago PAL-10S) prior to freezing. As we measure phthalate *metabolites* in urine rather than the parent phthalate diester prior to *in vivo* conversion, contamination from collection materials was not anticipated. Nonetheless, collection materials were screened for phthalate metabolite contamination. Field blanks collected alongside biospecimens were also analyzed for phthalate metabolites.

### Urinary phthalate biomarker analysis

Concentrations of fifteen metabolites of eleven phthalate diesters (Table 1) were measured at the Senator Frank R. Lautenberg Environmental Health Sciences Laboratory at the Icahn School of Medicine at Mount Sinai, using a method developed by the Centers for Disease Control and Prevention (S1 File).[47, 48] Urine sample preparation and analyses involved enzymatic deconjugation of phthalate metabolites from their glucuronidated form. The enzyme digested urine solutions were subjected to solid phase extraction with a reversed phase polymeric sorbent (Strata-X, Phenomenex), and extracts were analyzed using a Shimadzu Nexera XR UHPLC coupled with AB Sciex 6500 triple quadruple mass spectrometer. Target analytes were chromatographically separated on a Kinetex^®^ 2.6 μm Biphenyl column in a mobile phase consisting of acetonitrile and 0.1% acetic acid in gradient elution mode, ionized with an electrospray ionization source operating in negative mode, and quantified in multiple reaction monitoring mode. Samples, reagent blanks, and quality control (QC) materials were analyzed in random order and processed identically. QC material consisted of a pooled urine matrix spiked with low, medium, and high levels of target analytes. The National Institute of Standards and Technology reference material 3673 Organic Contaminants in Non-Smokers' Urine was analyzed along with the study samples to ensure the accuracy and reliability of the data.

**Table 1:** Phthalate diesters and corresponding metabolites.

	Phthalate Diester	Monoester Metabolite(s)
Low Molecular Weight Phthalates		
	Dimethyl phthalate (DMP)	Monomethyl phthalate (MMP)
	Diethyl phthalate (DEP)	Monoethyl phthalate (MEP)
	Di-iso-propyl phthalate (DiPrP)	Mono-iso-propyl phthalate (MiPP)
	Di-*n*-pentyl phthalate (DPeP)	Mono-*n*-pentyl phthalate (MnPP)
	Butylbenzyl phthalate (BBzP)	Monobenzyl phthalate (MBzP)
	Di-*n*-butyl phthalate (DnBP)	Mono-*n*-butyl phthalate (MnBP)
	Di-iso-butyl phthalate (DiBP)	Mono-iso-butyl phthalate (MiBP)
High Molecular Weight Phthalates		
	Di-*n*-octyl phthalate (DOP)	Mono(3-carboxypropyl) phthalate (MCPP)
		Mono-*n*-octyl phthalate (MOP)
	Di(2-ethylhexyl) phthalate (DEHP)	Mono(2-ethylhexyl) phthalate (MEHP)
		Mono(2-ethyl-5-hydroxyhexyl) phthalate (MEHHP)
		Mono(2-ethyl-5-oxohexyl) phthalate (MEOHP)
		Mono(2-ethyl-5-carboxypentyl) phthalate (MECPP)
		Mono(2-carbox-hexyl) phthalate (MCHP)
	Di-iso-nonyl phthalate (DiNP)	Mono-iso-nonyl phthalate (MiNP)

### Neurobehavioral assessment

The NICU Network Neurobehavioral Scale (NNNS), a standardized exam of infant neurobehavior, motor function, and stress response,[49–54] was administered by a certified examiner before NICU discharge at 33–37 weeks PMA. NNNS performance is reported as 13 summary scores (S2 File) of the newborn’s motor and behavioral performance and maturity and has been associated with motor, cognitive, and behavioral function in later childhood.[55] It is an established method for early detection of attention and motor deficits in preterm and toxin-exposed populations.[56–58]

### Statistical approach

Each measured biomarker level was adjusted for urinary dilution with urine specific gravity using the following adjustment: *Concentration(SG normalized) = Concentration(specimen) • (SG[reference]– 1)/(SG[specimen]– 1)* where *SG[reference]* was the median specific gravity for all specimens analyzed. The geometric mean per participant of biomarker levels from all specimens collected during the hospitalization was used for regression analyses of association with neurodevelopmental performance. Individual specimen biomarker levels were used in analyses aimed at medical equipment exposure classification.

### Covariate selection for regression analyses

We created a directed acyclic graph (DAG, DAGitty.net[59, 60]; S1 Fig) to identify demographic, socioeconomic, and clinical confounding variables for inclusion in our models. We tested numerous factors in our model that have been associated with neurodevelopmental outcomes of preterm infants including gestational age and size at birth, specific medical comorbidities before and after birth, nutritional experience (breast milk versus formula) and growth in the NICU, episodes of infection, need for surgery, etc. We found the following concepts relevant to reduce confounding: infant gender, gestational age at birth, status as a small for gestational age, severity of illness at birth, and NICU-based medical morbidity. We initially planned to use the updated Clinical Risk Index for Babies (CRIB II) score[61] as a proxy for severity of illness at birth. The CRIB II is a calculated score based on clinical data available within the first 24 hours of life developed to predict initial risk of mortality amongst low birth weight infants. The CRIB II includes birth weight, gestational age, infant gender, and arterial blood gas measurement of base deficit, a measure of metabolic acidosis. After reviewing our DAG, we chose to include the largest base deficit measured in the first 12 hours of life rather than the CRIB II score to account for illness severity at birth. This reduced the risk of over-specification in the model as the CRIB II score incorporates gestational age, birth weight, and appropriateness of birth weight, each of which was identified by our DAG for inclusion in models as a distinct variable. We created a composite dichotomous variable of NICU-based morbidity that indicated whether the infant experienced any of the following complications of prematurity: necrotizing enterocolitis, culture-proven sepsis, stage II-IV retinopathy of prematurity, grade II-IV intraventricular hemorrhage, or bronchopulmonary dysplasia defined as the need for respiratory support at 36 weeks PMA. A participant was considered to have experienced a significant NICU-based morbidity if he/she carried one or more than one of the specified complications of prematurity.

### Multivariable linear regression

Linear regression analysis was performed to identify the relationship between the sum of the geometric mean of DEHP metabolites (∑DEHP = [MEHP]+[MEHHP]+[MEOHP]+[MECPP]+[MCHP]) and NNNS summary scales adjusted for covariates. We expressed DEHP molar sum results in ng/mL for convenience of interpretation, using the molecular weight of MEHP (278g/mol) as a molecular weight estimate per Teitelbaum[62] and Wolff.[63] We used the Holm-Bonferroni method to address the problem of multiple comparisons.

### Weighted quantile sum (WQS) regression

As the complex NICU environment conveys exposure to mixtures of phthalates rather than exposure to single phthalate diesters in isolation, WQS regression[64] was performed to identify the relationship between mixtures of phthalate metabolites and performance on each summary scale of the NNNS. Using the geometric mean of concentration-adjusted biomarker measurements for each study infant, we used WQS regression to create empirically-weighted indices that identified “bad actors” based on non-negligible weights and yielded the association between the empirically-weighted exposure index and each summary scale outcome.[64, 65] WQS is conducted in two steps: (i) estimating a weighted index of standardized concentrations (e.g., scored into quantiles) in a nonlinear model with a link function to accommodate continuous, binary, or count data across 100 bootstrap samples; and (ii) testing for the significance of the constructed WQS in a generalized linear model of the outcome. A test for significance is a test for a mixture effect—which may be sub-threshold for individual components—in the direction associated with the parameter estimate; detection of a signal in the opposite direction is possible by estimating the weighted index with a constraint on the parameter estimate to be <0 or >0. These constraints, including constraints that the weights are between 0 and 1 and sum to 1, reduce ill-conditioning due to the complex correlations among the components, and are robust to correlation patterns in terms of sensitivity and specificity for identifying bad actors[64]. Bi-directional tests were conducted in all WQS analyses. WQS regression analyses included all monoesters measured above the LOD in > 85% of specimens collected and were adjusted for the same covariates as the multivariable linear regression analysis. We again used the Holm-Bonferroni method to address the problem of multiple comparisons.

### Assessment of medical equipment exposure

From enrollment until NICU discharge, trained study staff kept daily inventory of medical equipment exposure for each infant. Following Green *et al*.,[27] we categorized patient exposure to medical equipment likely to convey DEHP exposure at the time of urine specimen collection as low, medium, or high. Low DEHP exposure infants did not receive respiratory support and were fed enterally; i.e., they were managed in the NICU environment but had only minimal exposure to indwelling potentially DEHP-leaching plastic medical equipment. Medium-DEHP exposure infants received non-invasive respiratory support via nasal prongs, enteral feedings and/or gastric decompression via an indwelling gavage tube, and/or intravenous hyperalimentation via indwelling central catheter. High-DEHP exposure infants received invasive respiratory support via endotracheal tube, intravenous hyperalimentation via indwelling central catheter, and gastric decompression via an indwelling gavage tube. These high-exposure infants experienced the most intensive NICU therapy as they demonstrated the most significant illness at the time of specimen collection. It is worth noting, however, that medium- and high- exposure infants were distinguished more by the type of respiratory equipment they were exposed to rather than the number of separate pieces of equipment or the duration of exposure. For example, an infant managed with nasal support would be classified as “medium exposure” while an intubated infant would be classified as “high exposure” even if both infants had that single respiratory source of plastic exposure and identical central line and orogastric tube sources. Although the equipment exposure categories of Green *et al*. raises the possibility of misclassification between the medium and high exposure groups, we chose to use these established groupings to allow comparison to the existing, if sparse, literature on NICU-based phthalate exposure.

## Results

Eighty-one premature infants were enrolled in phase I of NICU-HEALTH. The 64 who survived, had at least one urine specimen analyzed for phthalate monoesters, and had NNNS performed prior to NICU discharge are the cohort evaluated in this study. Pertinent demographic characteristics are presented in Table 2. Due to the diversity of disease states found in the VLBW population, this study cohort experienced an anticipated variety of NICU care with the potential to convey differential phthalate exposure.[27, 66] The study cohort was similar to the parent NICU-HEALTH cohort in all characteristics evaluated.

**Table 2:** Demographic characteristics of participating infants.

		Mean ± STD, Number (%), or Median (IQR)	
		Study Cohort (n = 64)	*All* NICU-HEALTH phase I Participants (n = 81)
Demographic factors			
	Private insurance (#, %, vs Medicaid)	49 (76.6%)	63 (77.8%)
	Racial/ethnic minority	31 (48.4%)	40 (49.4%)
	White	37 (57.8%)	47 (58.0%)
	Black	18 (28.1%)	24 (29.6%)
	Asian	6 (9.4%)	7 (8.6%)
	Hispanic	5 (7.8%)	6 (7.4%)
	Male infant gender	31 (48.4%)	39 (48.1%)
Maternal/antenatal factors			
	Maternal parity at index pregnancy	2 (1, 3)	2 (1, 3)
	Maternal age at delivery, years	33.1 ± 7.3	33.0 ± 7.0
	Mulitple gestation	38 (59.4%)	44 (54.3%)
	Antenatal steroids	63 (98.4%)	80 (98.8%)
	Antenatal magnesium sulfate	62 (96.9%)	77 (95.1%)
	PPROM	24 (37.5%)	30 (37.0%)
	IUGR	10 (15.6%)	16 (19.8%)
	Pre-eclampsia	12 (18.8%)	18 (22.2%)
	Assisted reproductive technology	28 (43.8%)	32 (39.5%)
	Cesarean delivery	57 (89.0%)	72 (88.9%)
Birth Characteristics			
	Gestational age at birth (weeks)	28.3 ± 2.2	28.4 ± 2.2
	Birth weight (g)	1080 ± 270	1090 ± 270
	SGA	4 (6.3%)	7 (8.6%)
	1 min Apgar	8 (5, 9)	8 (6, 9)
	5 min Apgar	9 (8, 9)	9 (8, 9)
	CRIB II Score	8 (6, 11)	8 (6, 11)
Morbidities of Prematurity			
	Hypotension requiring pressors	21 (32.8%)	23 (28.4%)
	Bronchopulmonary dysplasia	25 (39.1%)	28 (34.6%)
	Necrotizing enterocolitis	1 (1.6%)	2 (2.5%)
	Culture proven sepsis	11 (17.2%)	14 (17.3%)
	Retinopathy of prematurity stage 2–4	12 (18.8%)	12 (14.8%)
	Intraventricular hemorrhage grade II-IV	7 (10.9%)	7 (8.6%)
	PMA at NNNS assessment (weeks)	34.6 ± 0.6	N/A

One hundred sixty four urine specimens were analyzed for 15 phthalate monoester metabolites. Five monoesters, MiPP, MCHP, MnPP, MiNP, and MOP, were measured above the LOD in < 15% of specimens and were not included in statistical analyses. Specific gravity-adjusted urinary phthalate concentrations are presented in Table 3.

**Table 3:** Distribution of specific gravity-adjusted phthalate concentrations measured in the cohort (ng/mL).

	LOD (ng/mL)	N (%) > LOD	5^th^ percentile	25^th^ percentile	median	75^th^ percentile	95^th^ percentile	min	max
MMP	0.10	100	1.37	2.82	6.64	11.91	25.59	0.48	67.32
MCPP	0.10	89	0.07	0.88	2.16	3.62	9.4	0.05	18.88
MEP	0.10	100	3.09	10.26	21.13	36.73	111.35	0.83	171.32
MECPP	0.10	100	3.24	24.63	49.72	79.27	201.39	0.2	304.95
MNBP	0.10	100	1.52	17.08	36.82	60.41	170.85	0.27	190.91
MiBP	0.10	100	1.09	6.33	15.26	26.87	66.26	0.28	293.83
MEHHP	0.10	100	1.67	6.01	11.84	27.98	99.04	0.56	164.58
MBzP	0.10	99	0.73	12.55	27.37	53.56	168.34	0.17	289.41
MEHP	0.10	100	1.19	3.55	7.12	11.68	28.81	0.5	72.39
MEOHP	0.10	100	1.27	6.18	11.95	24.46	70.47	0.55	148.57

Assessment with the NNNS was completed prior to NICU discharge. Descriptive statistics of our cohort’s performance alongside available comparative populations[67] are provided in Table 4. Of note, truly normative data for preterm infants are not available. Reference preterm infants were drawn from the Maternal Lifestyle Study,[52] a large prospective longitudinal study of prenatal drug exposure and child outcome. Compared to the reference populations, our cohort differed significantly in performance in Excitability and Arousal. Our cohort was both less excitable and exhibited less arousal than the reference groups. This could be due to our cohort’s relative immaturity compared to healthy term infants evaluated at term, and due to our cohort’s lack of exposure to illicit drugs compared to the reference preterm group. Infants demonstrating neonatal abstinence syndrome following *in utero* drug exposure are known to be more easily excitable and with a baseline higher level of arousal than non-withdrawing populations.

**Table 4:** NNNS performance.

NNNS Summary Scale	Mean ± STD in the NICU-HEALTH phase I cohort	Mean ± STD for reference preterm infants GA 24–32 weeks	Mean ± STD for reference well term infants
Habituation	6.89 ± 2.12	7.06 ± 1.96	7.91 ± 1.14
Attention	4.15 ± 0.70	5.30 ± 1.39	5.30 ± 1.04
Handling	0.05 ± 0.10	0.50 ± 0.28	0.27 ± 0.27
Non-Optimal Reflexes	4.49 ± 1.53	5.05 ± 2.21	4.32 ± 1.73
Regulation	5.63 ± 0.50	5.01 ± 0.86	5.00 ± 0.82
Excitability	1.04 ± 1.10	4.59 ± 2.76	4.23 ± 2.43
Quality of Movement	4.87 ± 0.39	4.27 ± 0.78	3.81 ± 0.78
Stress/Abstinence	0.08 ± 0.04	0.20 ± 0.09	0.15 ± 0.05
Arousal	2.98 ± 0.25	4.34 ± 0.71	4.16 ± 0.81
Lethargy	6.09 ± 1.98	3.50 ± 2.00	6.32 ± 3.24
Hypertonicity	0.01 ± 0.12	0.77 ± 1.13	0.07 ± 0.26
Hypotonicity	0.01 ± 0.14	0.26 ± 0.59	0.55 ± 0.76
Asymmetric Reflexes	1.60 ± 4.10	0.9 ± 1.16	1.93 ± 1.33

Adjusted multivariable linear regression analysis demonstrated small but significant associations between ∑DEHP exposure and multiple summary scales of the NNNS (Table 5). ∑DEHP was positively associated with scores on the Attention and Regulation summary scales. Higher Attention and Regulation scores indicate *improved* performance. After Holm-Bonferroni correction, the association between ∑DEHP and Attention remained significant.

**Table 5:** Adjusted multivariable linear regression of the association between ∑DEHP and NNNS summary scales^a^.

	Direction of Improved Performance^b^	β^c^	95% Confidence Interval	P value	Holm-Bonferroni P value
Habituation	Positive	-0.58	-1.25, 0.11	0.10	0.50
Attention	Positive	0.22	0.11, 0.36	0.002*	0.01*
Non-Optimal Reflexes	Negative	-0.19	-0.44, 0.08	0.16	0.64
Regulation	Positive	0.08	0, 0.17	0.03*	0.18
Excitability	Positive	-0.14	-0.33, 0.06	0.19	0.64
Quality of Movement	Positive	0.03	-0.06, 0.08	0.53	1
Lethargy	**–**	-0.08	-0.44, 0.25	0.59	1

^a^ Models included the following covariates: infant gender; gestational age at birth, status as small for gestational age, largest base deficit in the first 24-hours, and a composite score of NICU-based morbidity.

^b^ Direction of improved performance refers to the optimal performance on each scale. For example, more mature infants or those with better habituation demonstrate higher scores on the Habituation scale. More mature infants or those with better attention demonstrate higher scores on the Attention scale. For the scales Arousal and Lethargy, scores at either extreme (high or low) are abnormal. In most cases of a significant association, the direction of change in NNNS performance represents an improvement in performance on that summary scale.

^c^ β values represents the point change in NNNS summary scale score per 10 ng/mL increase in urinary ∑DEHP.

* Significant with p < 0.05.

WQS regression revealed significant associations between specific mixtures of phthalate biomarkers and performance on the NNNS summary scales of Attention, Handling, Non-Optimal Reflexes, Regulation, Excitability, and Habituation (Table 6). After Holm-Bonferroni correction, the association between specific phthalate mixtures and performance on the NNNS summary scales of Attention, Handling, and Non-Optimal Reflexes remained significant while the association with Excitability approached significance. As expected, weighted indices were outcome-specific; i.e., each outcome demonstrated a unique clinically important mixture of phthalate metabolites. Mixtures of phthalates were associated with *improvement* in summary scale performance in all subscales except habituation. Habituation summary scale assessments were only available for 18/64 (28%) infants due to the specific resting state requirements of habituation maneuvers. Other NNNS summary scales were based on the full cohort. Certain phthalate monoesters—notably MEOHP—were implicated as clinically important to performance on multiple summary scales.

**Table 6:** Association of outcome-specific weighted indices of phthalate biomarkers with summary scale performance on the NICU Network Neurobehavioral Scale^a^.

NNNS Summary Scale	Direction of Improved Performance^b^	β^c^	95% Confidence Interval	P value	Holm-Bonferroni P value	Predominant Index Monoesters	WQS Index Weight^d^
Habituation	Positive	-0.024	-0.046, -0.002	0.04*	0.2	MECPP	0.23
						MEP	0.18
						MMP	0.17
Attention	Positive	0.20	0.06, 0.34	0.004*	0.04*	MEOHP	0.47
						MEHHP	0.45
Handling	Negative	-1.09	-1.86, -0.32	0.006*	0.048*	MECPP	0.23
						MEP	0.18
						MMP	0.17
Non-Optimal Reflexes	Negative	-0.42	-0.71, -0.13	0.005*	0.045*	MEOHP	0.40
						MCPP	0.22
						MEP	0.18
Regulation	Positive	0.11	0.02, 0.20	0.02*	0.12	MEOHP	0.70
						MEP	0.16
Excitability	Negative	-0.29	-0.53, 0.06	0.01*	0.07	MEOHP	0.43
						MCPP	0.27
						MEP	0.19
Quality of Movement	Positive	0.04	-0.03, 0.10	0.30	0.78	MEOHP	0.76
Stress	Negative	0.006	-0.002, 0.01	0.15	0.6	MBzP	0.52
						MCPP	0.16
Arousal	**–**	0.02	-0.03, 0.07	0.44	0.78	MEHHP	0.42
						MiBP	0.31
Lethargy	**–**	-0.12	-0.53, 0.28	0.26	0.78	MCPP	0.34
						MECPP	0.34

^a^ Models included the following covariates: infant gender; gestational age at birth, status as small for gestational age, largest base deficit in the first 24-hours, and a composite score of NICU-based morbidity.

^b^ Direction of improved performance refers to the optimal performance on each scale. For example, more mature infants or those with better habituation demonstrate higher scores on the Habituation scale. More mature infants or those with better attention demonstrate higher scores on the Attention scale. For the scales Arousal and Lethargy, scores at either extreme (high or low) are abnormal. In most cases of a significant association, the direction of change in NNNS performance represents an improvement in performance on that summary scale.

^c^ Increasing absolute values of the WQS β estimate reflect increasingly strong linear associations between exposure mixtures and health outcomes.

^d^ Index monoesters contributing >15% of effect are listed.

* Significant with p < 0.05.

**Fig 1:**
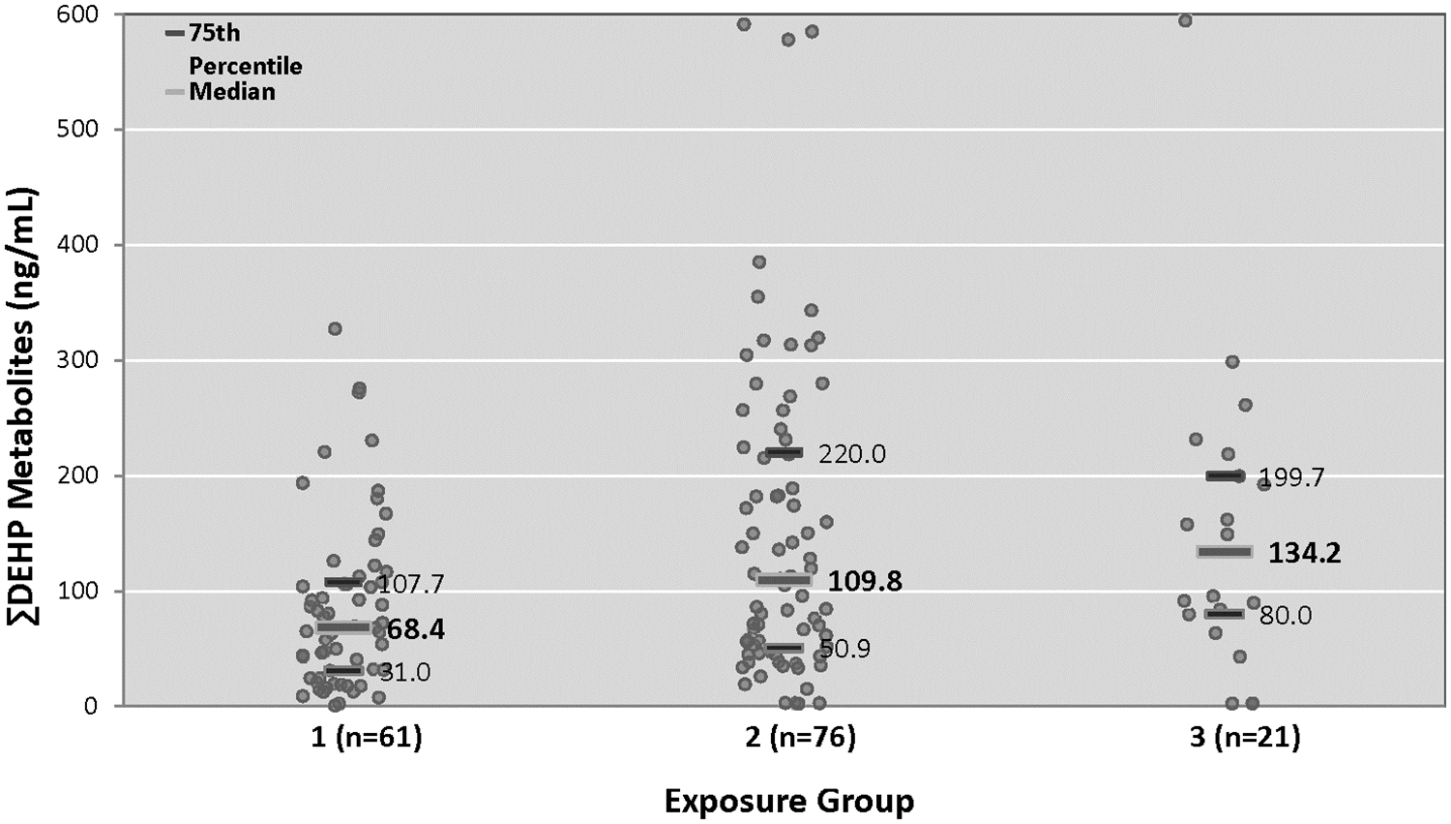
∑DEHP metabolites by medical equipment exposure group. Molar ∑DEHP metabolites represented in ng/mL using the molecular weight of MEHP for conversion. Twenty-fifth percentile, median, and 75^th^ percentile values in ng/mL are indicated for each medical equipment exposure group. Groups 1–3 are as defined in the Methods section, with group 1 representing the lowest exposure to medical equipment and group 3 representing the highest acuity, highest intensity equipment exposure.

Based on daily assessments of clinical equipment exposure and the known timing of urine specimen collection, we were able to link 158 individual urine specimens with a distinct medical exposure category (Fig 1). Exposure to progressively more invasive medical equipment was associated with progressively higher median urinary ∑DEHP levels. Median urinary ∑DEHP levels in infants in the low-, medium-, and high-DEHP exposure groups were 68.5, 109.8, and 134.3 ng/mL respectively. Interestingly, medical equipment exposure group 2, those marked by non-invasive respiratory support, had the highest 75th percentile values of ∑DEHP exposure.

## Discussion

This study is the first to relate NICU-based phthalate exposure to neurobehavioral outcome. As a biomarker-based prospective study designed from its inception as an environmental health cohort of premature infants, it is unique in its population and approach. The primary finding, that NICU-based phthalate exposure was associated with *improved* performance on specific NNNS summary scales was unexpected, but consistent across analyses focused only on DEHP metabolites and on agnostically derived phthalate mixtures.

Phthalate metabolite levels in the first phase of NICU-HEALTH were lower than those previously reported in NICU patients.[26, 28] This may be due to changes in manufacturing to reduce DEHP exposure in vulnerable infants during the decade separating older studies from ours, variability in phthalate metabolism presented by our exclusively preterm population, reductions in measurement contamination over the intervening decade,[68] or changes in NICU care. Phthalate exposure in our study was comparable to or higher than clinically-relevant exposure in a contemporaneous non-hospitalized full-term cohort,[69] and to *in utero* exposure associated with adverse neurodevelopmental outcomes in recent term birth cohorts.[41, 45, 70]

Based on studies of *in utero* phthalate exposure in term children and behavioral outcomes in middle childhood,[33, 34, 36, 37, 42–45] we expected that elevated phthalate exposure would predict worse behavioral performance, particularly in domains related to attention and social reactivity. Surprisingly, our preterm infants who demonstrated higher levels of specific mixtures of phthalate metabolites performed better than expected on multiple NNNS summary scales. We had anticipated the opposite result, not only because prior studies showed associations between phthalate exposure and adverse neurodevelopmental outcomes,[33–45] but also because the sickest, most premature infants require a higher intensity of medical care that would be expected to have the highest phthalate exposure.[24–26, 28, 71] These sickest and most premature infants typically demonstrate worse neurobehavioral performance thought to be related to severity of illness. Although the intubated infants in our study classified as “high” medical equipment exposure indicative of more severe illness did have the highest median DEHP biomarker levels (Fig 1), we found that those infants demonstrating the highest level of clinically-relevant phthalate exposure actually performed *better* on multiple NNNS summary scales.

Although unexpected, our findings are biologically plausible and may provide insight into the sometimes inconsistent data on the relationship between phthalate exposure during early development and neurodevelopmental outcomes. Our outcome measure, the NNNS, is a measure of neurobehavioral *development*, not a measure of static cognitive ability or behavior. As NNNS performance improves with maturity, improved summary scale scores can be interpreted as either improved ability compared to PMA-matched peers, or attainment of neurodevelopmental milestones earlier than expected. Commonly proposed mechanisms of action of phthalate neurotoxicity involve endocrine disruption via interference with androgen synthesis and thyroid function—two pathways known to be critically important to neurodevelopment. Additionally, phthalate exposure is linked to epigenetic modification which could alter neurodevelopmental velocity during times of neuronal plasticity.[72–74] If phthalates prevented the development of normal inhibitory responses that protect the nervous system from excess stimulation, infants with greater phthalate exposure would demonstrate elements of the pattern seen in our cohort: hyper-attention, hyperreflexia, and difficulty being soothed. The sensitivity of premature infants to hyperstimulation has been long recognized; multiple established care practices (low lighting, covered incubators, clustered cares) aimed at reducing environmental stimulation have been shown to improve preterm brain development and motor function.[75]

Epidemiological studies in other fields have demonstrated the relationship between environmental exposures during the third trimester neurodevelopmental window and more rapid behavioral maturation. Specifically, Posner *et al*.[76] found that term-born infants exposed to elevated maternal stress in late-pregnancy demonstrated behavioral and neuroanatomical phenotypes expected for children of an older age. As attentional performance in young children generally improves with age, these children scored unexpectedly *well* on neurobehavioral testing early in life. It was hypothesized that the stressful *in utero* environment provoked rapid maturation as a protective mechanism for an anticipated stressful postnatal environment. When followed into middle childhood, these children transition to a phenotype of inattention and hyperactivity, thus demonstrating the widely recognized association between prenatal exposure to stress and poor behavioral outcome in childhood.[77–80] The “hyper-attention” of infancy became maladaptive with age.

Our study has a number of strengths. It was designed to address limitations of the few other biomarker-based studies of NICU-based phthalate exposure in the literature,[26–28] which were limited by cross-sectional design, heterogeneous enrollment without attention to factors likely to affect phthalate metabolism (e.g., gestational age, PMA), and absence of prospectively-designed clinical outcome assessments. All study participants were cared for in the same unit using a standardized care approach. Although individual care practices may have varied somewhat, the level of medical intervention for a given clinical presentation was fairly uniform in this single ICU population, reducing the risk of confounding by provision of medical care. Our statistical approach focused on mixtures is novel and highly relevant to studies of NICU-based phthalate exposure. NICU inpatients are exposed to phthalate mixtures through the complex materials used concurrently in NICU care; respiratory circuits, intravenous equipment, enteral feeding supplies, and incubators are likely vehicles of phthalate exposure.[66] Our finding that clinically significant phthalate exposure was associated with *improved* NNNS performance reduced concerns about confounding by indication.

Our study does have some limitations. Our patient population, very low birth weight infants cared for at a level IV academic regional perinatal center, may not be representative of the entire NICU population. As data on “typical” community-based exposure to phthalates in early infancy in non-NICU and non-preterm populations is not available (the youngest patients with phthalate biomarker measures in the National Health and Nutrition Examination Survey, for example, are age 6), comparisons to a relevant non-NICU group is not possible. The half-life of phthalate diester metabolism in preterm infants is not well characterized, but is known to vary between 4 and 24 hours in adults.[81–84] This short half-life indicates that phthalate levels likely reflect source exposure in the preceding day or two. Thus, individual specimen data was used to link biomarker data to potential source categories based on medical equipment exposure immediately preceding specimen collection. As is common in environmental health research, we used the geometric mean of serial participant biomarker levels to estimate changing exposure over the course of the NICU stay. If a different estimate (e.g. peak, arithmetic mean, median) is actually the best estimate of clinically-relevant phthalate exposure during the NICU stay, we may have under- or over-estimated exposure for patients with wider ranges of biomarker levels. Although used in other NICU[56, 85, 86] and environmental health[41, 87, 88] studies, the NNNS is a very early outcome measure. Although most participants underwent NNNS assessment at 34 to 35 weeks PMA (mean 34.6 ± 0.6 weeks), there was some variation in the timing of evaluation such that the range of evaluation ages was PMA 33–37 weeks. Covariates were selected using rigorous and accepted methodology; nonetheless it is possible that confounders not considered or not measured could impact results. For example, we did not include PMA at NNNS assessment as a covariate in our model as its inclusion was not supported by our DAG. Given the limited sample size in this study, we were unable to consider sex-specific associations that have been reported in some of the previous studies of phthalates and neurobehavioral outcomes. Finally, our total data set was not large enough to eliminate the risk of over-fitting; testing was done on the same data as WQS weight assignments because our sample size was inadequate to support cross-validation. These deficiencies will be addressed in ongoing larger studies.

Although many NICUs—including ours—are increasingly labeled “phthalate-free” to signify the use of DEHP-free equipment whenever available, replacements for DEHP are often other phthalates[89] or non-phthalate organic chemicals that may be neuro-active but not included in our current biomarker testing panel. Nonetheless, modern NICUs convey significant DEHP and non-DEHP phthalate exposure through the many plastic medical materials that are not available in phthalate-free form.[66]

## Conclusions

In this first phase of a prospective cohort study of the neurodevelopmental impact of NICU-based phthalate exposure on very low birth weight infants, specific mixtures of phthalate biomarkers were associated with *improved* attention and social response on the NICU Network Neurobehavioral Scale. The long-term impact of this association between phthalate exposure and neurobehavior needs to be evaluated as our preterm cohort ages.

## Supporting information

S1 File Analytical method: Urinary phthalates metabolites. (DOCX)

S2 File Clinical interpretation of relevant NNNS summary scores. (DOCX)

S1 Fig Directed acyclic graph depicting covariate selection. Covariates included in the model were unique elements of the CRIB-II score, gestational age at birth, a variable representing medical illness in the NICU, and gender.
(TIF)
