# Supplementary material for: Neonatal intensive care unit phthalate exposure and preterm infant neurobehavioral performance
Source: PLoS One. 2018 Mar 5;13(3):e0193835. doi: 10.1371/journal.pone.0193835 (PMC5837295; doi:10.1371/journal.pone.0193835)
Supplement: S1 File — (DOCX) [file pone.0193835.s001.docx]

**Supporting information 1**

**Analytical method: urinary phthalates metabolites**

**Standards and reagents**

The fifteen phthalate metabolites were monomethyl phthalate (MMP), monoethyl phthalate (MEP), mono-iso-propyl phthalate (MiPP), mono-n-pentyl phthalate (MnPP), monobenzyl phthalate (MBzP), mono-n-butyl phthalate (MnBP), mono-iso-butyl phthalate (MiBP), mono(3-carboxypropyl) phthalate (MCPP), mono-n-octyl phthalate (MOP), mono(2-ethylhexyl) phthalate (MEHP), mono(2-ethyl-5-hydroxyhexyl) phthalate (MEHHP), mono(2-ethyl-5-oxohexyl) phthalate (MEOHP), mono(2-ethyl-5-carboxypentyl) phthalate (MECPP), mono(2-carbox-hexyl) phthalate (MCHP), and mono-iso-nonyl phthalate (MiNP). The eleven isotopically labeled internal standards were MMP-^13^*C*_4_, MEP-^13^*C*_4_, MBzP-^13^*C*_4_, MnBP-^13^*C*_4_, MCPP-^13^*C*_4_, MOP-^13^*C*_4_, MEHP-^13^*C*_4_, MEHHP-^13^*C*_4_, MEOHP-^13^*C*_4_, MECCP-^13^*C*_4_, and MiNP-^13^*C*_4_. All analytical standards were purchased from Cambridge Isotope Laboratories (Cambridge, MA, USA). The working stock solutions of phthalate metabolites, internal standards and their mixtures were prepared at 100 mg/mL and 1 mg/mL in acetonitrile and stored at −20° C.

LC/MS grade acetonitrile, methanol, acetic acid, formic acid, and water were purchased from Fisher Scientific. High purity ammonium acetate, anhydrous sodium acetate, and phosphoric acid were purchased from Fisher Scientific. β-Glucuronidase from *Helix pomatia* was from Sigma-Aldrich (St. Louis, MO, USA). All the solvents and reagents were tested for phthalates metabolites contamination, and determined to be below limits of detection as determined by high performance liquid chromatography coupled with tandem mass spectrometry (LC–MS/MS) analysis.

**Urine sample preparation and pre-treatment**

Urine preparation for phthalates metabolites extraction was based on the CDC method (Silva *et al*. 2004; Silva *et al*. 2007) and with minor modifications from Guo *et al* (2011) and Dewalque *et al*. (2014). In brief, 0.5 mL urine was transferred to a 7 mL Pyrex glass tube and spiked with 25 μL of an internal standards mixture solution containing eleven isotopically labelled phthalate metabolites at a concentration of 50 ng/mL of each component. After being vortexed for 60 s, the urine sample was buffered with 200 μL of 1.0 M ammonium acetate solution at pH 4.5 adjusted with acetic acid, and 25 μL of β-glucuronidase from *Helix pomatia* (type H-2) was added. After being vortexed for another 60 s, the mixture was incubated overnight at 37 °C in a shaking water bath to hydrolyze the phthalates conjugates.

**Solid phase extraction**

SPE was performed using a 96-well plate format containing 30 mg of polymeric reversed phase SPE sorbent per well (Strata™-XL 100 µm, 30 mg/well) from Phenomenex (Torrance, CA, USA). The first step was to equilibrate the wells by adding 300 μL of acetonitrile and condition with 300 μL of 0.5% aqueous acetic acid. The second step was to acidify the urine samples with 50 μL acetic acid, centrifuged, and loaded onto the SPE well plate. Third, the wells were slowly washed with 600 μL of 0.5% aqueous acetic acid. Fourth, the phthalate metabolites were eluted twice with 600 μL of acetonitrile. Eluant was collected in 2mL glass inserts and evaporated to dryness under a nitrogen stream at 40 °C in a water bath, and reconstituted in 100 μL of a 30% aqueous acetonitrile with 0.1% acetic acid, homogenized, and analyzed by UPLC–MS/MS instrumentation operated in electrospray ionization in negative ion mode as described in the following sections.

**Liquid chromatography**

A Shimadzu Nexera XR UHPLC was used for the chromatographic separation of phthalates metabolites on a Kinetex biphenyl column (50 x 2.1 mm, 2.6µm, 100°A) from Phenomenex (Torrance, CA, USA). The mobile phase A consisted of 0.1% acetic acid in LC/MS grade water and mobile phase B was 0.1% acetic acid in LC/MS grade acetonitrile. A flow rate of 0.3 mL/min was used with the following gradient program: 0.0-1.0 min (5 % B), 2.0 min (20 % B), 3.0 min (40 % B), 7.0 min (50 % B), 7.0-10.0 min (50 % B), 11.0 min (90 % B), 11.0-12.0 min (90 % B), 12.1 min (5 % B), and 12.1-15.0 min (5 % B). The injection volume was 10 μL.

**Tandem mass spectrometry**

A Sciex 6500 triple quadruple mass spectrometer equipped with electrospray ionization (ESI) source (SCIEX, Framingham, MA, USA) was operated in negative ionization mode for the detection and quantitation of phthalates metabolites. Nitrogen was used as curtain and collision gas. Ion source and gas parameters were set to the following values: curtain gas flow = 20 psi; nebulizer gas (ion source gas 1) and heater gas (ion source gas 2) = 50 psi each; source temperature = 450ºC; and ion source voltage = -4500 V. The collision gas value was set to 8. Compound parameters such as declustering potential, entrance potential, and collision exit potential were set to -35, -10, and -13, respectively. In the mass spectrometer, multiple-reaction monitoring mode (MRM) was used for data acquisition of each target analyte. Most prominent ion transition was used for quantitation and the most intense second ion transition was used for confirmation. The collision energy was individually optimized by direct syringe infusion of each compound into the mass spectrometer.

The MRM transitions were 178.9 → 77.0, 106.9 for MMP, 193.0 → 77.0, 121.0 for MEP, 207.0 → 121.0, 135.0 for MiPP, 235.0 →85.0, 235.0 for MnPP, 225.0 → 107.0, 182.9 for MBzP, 221.0 →76.9, 177.0 for MnBP, 221.0 →77.0, 177.0 for MiBP, 251.0 →77.0, 103.0 for MCPP, 277.0 →77.0, 125.0 for MOP, 277.0 →121.7, 133.9 for MEHP, 293.0 → 77.0, 121.0 for MEHHP, 291.0 →121.0, 143.0 for MEOHP, 307.0 →121.0, 159.0 for MECPP, 247.0 → 77.0, 97.0 for MCHP, and 291.0 → 77.0, 141.0 for MiNP. The collision energies for respective -20eV and -20eV for MRM transitions were: -24eV and -14eV MMP, -22eV and -16eV for MEP, -20eV and -20eV MiPP, -20eV and -20eV MnPP, -28eV and -16eV for MBzP, -22eV and -14eV for MnBP, -20eV and -20eV MiBP, -20eV and -20eV MCPP, -20eV and -30eV MOP, -20eV and -20eV for MEHP, -38eV and -20eV MEHHP, -20eV and -20eV MEOHP, -20eV and -20eV MECPP, -20eV and -20eV MCHP, and -50eV and -30eV MiNP.

**Quantitation**

Phthalate metabolites were quantified using isotope-labelled internal standard calibration. The calibration curve was built on the peak area of the ion chromatogram of the quantitation transition of the individual analyte in the MRM mode. The concentration range of the calibration curve differed by the phthalate metabolite, but each of them were spiked with the same amount of respective isotope-labelled internal standard. Same concentration of the internal standard mixture was spiked in each urine sample. Internal standard calibration curve for each phthalate metabolite was based on plotting the ratio of the target ion are of the quantitation MRM transition to that of the ion area of the corresponding isotope-labelled internal standard against the spiked concentration of the target analyte (ng/mL) with 1/*x* weighing. For example, MMP-^13^*C*_4_ was used as the isotope-labelled internal standard for MMP in building the calibration curve. Similarly, MEP-^13^*C*_4_ was used for MEP, MiPP, and MnPP, MBzP-^13^*C*_4_ was used for MBzP, MnBP-^13^*C*_4_ was used for MnBP and MiBP, MCPP-^13^*C*_4_was used for MCPP, MOP-^13^*C*_4_ was used for MOP, MEHP-^13^*C*_4_ was used for MEHP, MEHHP-^13^*C*_4_ was used for MEHHP, MEOHP-^13^*C*_4_ was used for MEOHP, MECCP-^13^*C*_4_ was used for MECPP, MBzP-^13^*C*_4_ was used for MCHP, and MiNP-^13^*C*_4_ was used for MiNP.

**Method performance**

The limits of detection (LOD) for phthalate metabolites varied from 0.10 to 0.25 ng/mL. The limits of quantitation (LOQ) of all target analytes varied from 0.25 to 1.00 ng/mL. Isotope-labelled internal standard adjusted calibration curves were linear over the whole calibration range between 0.05 and 100 ng/mL for corresponding phthalate metabolite. NIST standard reference material (SRM) 3673 -Organic Contaminants in Non-Smokers' Urine, and a pooled urine matrix spiked with low, medium and high levels of individual target analytes were used as quality control samples. Individual batch runs were included with 20% quality control samples including duplicates, experimental, matrix spikes (low/medium/high), and NIST standard reference material (SRM 3673). The recoveries were all within 25% of the respective target values. Phthalate metabolites in the experimental blanks were below the respective LODs. Reproducibility (inter- and intra-day) was within 10% for all target analytes of interest. Randomly selected study samples were analyzed in duplicate randomly within and between batches, and the relative percent differences between duplicate results were within 20% for all analytes. Target analyte concentrations were reported in ng/mL of urine, which were later adjusted for urinary dilution using urine specific gravity information.
