# Supplementary material for: Neonatal intensive care unit phthalate exposure and preterm infant neurobehavioral performance
Source: PLoS One. 2018 Mar 5;13(3):e0193835. doi: 10.1371/journal.pone.0193835 (PMC5837295; doi:10.1371/journal.pone.0193835)
Supplement: S2 File — (DOCX) [file pone.0193835.s002.docx]

**Supporting information 2**

**Clinical interpretation of relevant NNNS summary scores**

[After Lester, *et al.*([51](#_ENREF_51))]

**Arousal**

This score relates the infant’s state of arousal reached throughout the examination including associated motor activity, whether and how quickly the infant becomes irritable, and the infant’s overall activity in response to handling. A high score indicates an infant who fusses and cries easily in response to stimulation and who is highly active even when left alone.

**Attention**

This scale indicates an infant’s response to specific directed auditory and visual stimulation. Infants with high scores on this scale show the ability to track the stimulus and maintain alertness through an extended task. Low scores indicate either inability to track or brief response.

**Excitability**

This scale measures the state-related level of arousal over the course of the whole examination. It reflects the peak level of excitement reached by the infant, the amount of stimulation required to reach this peak, the ability of the infant to maintain this peak, and the ability of the infant to react to the outside world during the peak level of excitement.

**Habituation**

This scale measures the capacity of the infant to maintain a sleep state by progressively “tuning out” a series of proscribed stimuli, following an initial response. Higher scores are indicative of better (more rapid) habituation. Low scores indicate the infant’s delay in inhibiting the response, resulting in disturbed sleep.

**Handling**

This score describes the number and types of maneuvers that were necessary to keep the infant in the appropriate state (awake, alert, calm) to administer specific auditory and visual stimulation tasks. These include the amount and type of handling needed to arouse the infant from a drowsy to an alert state or to soothe the infant from an irritable or crying state. High scores indicate infants who need substantial input from the examiner to maintain an alert and calm state.

**Lethargy**

This score relates the infant’s state of sleepiness during the examination. The NNNS examination is typically initiated on a sleeping infant, 30-60 minutes prior to scheduled feeding. Infants will wake with varying rapidity and completeness throughout the exam. In the face of identical stimulation, some infants will rapidly wake such that habituation cannot be fully evaluated, while others will remain quite sleepy through much of the exam.

**Non-optimal reflexes**

This score represents a count of non-optimal (hyper-reflexive or weak) reflexes.

**Quality of movement**

This scale is a measure of motor control including gross motor smoothness and maturity, as well as startles and tremors. High scores (good quality of movement) indicate infants with smoother, more mature movements and less jitteriness.

**Regulation**

This score combines physiological, motor, and attentional activation, with the ease or difficulty on the part of the examiner, of soothing the infant. It also incorporates the infant’s capacity to self-soothe, and indicates the infant’s ability to cope with the demands of the examination, with higher scores indicating better regulation/coping.

**Stress**

This scale includes physiological signs of general stress and of drug withdrawal (abstinence). High scores are most consistent with significant symptoms of withdrawal from opioids.
