# Supplementary figures and images for: Neonatal intensive care unit phthalate exposure and preterm infant neurobehavioral performance
Source: PLoS One. 2018 Mar 5;13(3):e0193835. doi: 10.1371/journal.pone.0193835 (PMC5837295; doi:10.1371/journal.pone.0193835)

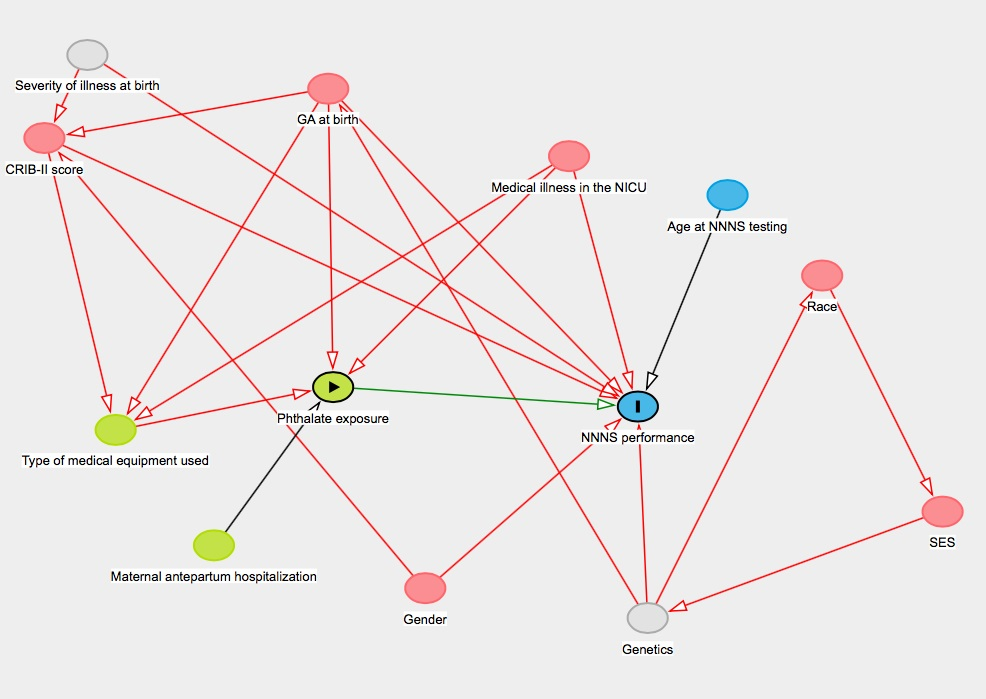

Supplement: S1 Fig — Covariates included in the model were unique elements of the CRIB-II score, gestational age at birth, a variable representing medical illness in the NICU, and gender. (TIF) [file pone.0193835.s003.tif]
